# Contraction of the Orifice of the Pulmonary Artery without Tuberculosis

**Published:** 1882-10

**Authors:** 


### CONTRACTION OF THE ORIFICE OF THE PULMONARY ARTERY WITHOUT TUBERCULOSIS.

The patient, twenty-one years of age, had been treated for varicocele, and he had been minutely examined on account of certain curious phenomena. The testicles, rather distinctly palpable, were enveloped in a soft mass, formed by the venous plexus of the spermatic cord, which had enlarged to a considerable size. The dilatation extended also to the veins of the scrotal integument. The face of the patient was cyanotic; on the abdomen and the lower extremities there were strongly developed venous irradiations, forming on certain parts veritable bluish sacs. All these phenomena had progressively developed, the patient noticing them first five or six years ago. There was no rheumatism, but the patient had sometimes violent palpitation of the heart. Four days before his death he acquired an angina by taking cold, and erysipelas, probably of herpetic character, set in. He died suddenly, without any other symptoms. The autopsy showed the right ventricle of the heart dilated; the orifice of the pulmonary artery very much contracted. There was no endocarditis, but the liver was affected with hypertrophic cirrhosis.—*Progrès Médical.*

